# MALBAC-based chromosomal imbalance analysis: a novel technique enabling effective non-invasive diagnosis and monitoring of bladder cancer

**DOI:** 10.1186/s12885-018-4571-7

**Published:** 2018-06-15

**Authors:** Hao Liu, Wang He, Bo Wang, Kewei Xu, Jinli Han, Junjiong Zheng, Jun Ren, Lin Shao, Shiping Bo, Sijia Lu, Tianxin Lin, Jian Huang

**Affiliations:** 1 Department of Urology, Sun Yat-Sen Memorial Hospital, Sun Yat-Sen University, 107 Yanjiangxi Road, Guangzhou, China; 2 Guangdong Provincial Key Laboratory of Malignant Tumour Epigenetics and Gene Regulation, Sun Yat-Sen Memorial Hospital, Sun Yat-Sen University, 107 Yanjiangxi Road, Guangzhou, China; 3 Department of Clinical Research, Yikon Genomics, 1698 Wangyuan Road, Building #26, Fengxian District, Shanghai, 201400 China

**Keywords:** Bladder Cancer, CNV, MALBAC, NGS, Chromosomal imbalance analysis

## Abstract

**Background:**

The gold standard for bladder cancer detection is cystoscopy, which is an invasive procedure that causes discomfort in patients. The currently available non-invasive approaches either show limited sensitivity in low-grade tumours or possess unsatisfying specificity. The aim of the present study is to develop a new non-invasive strategy based on chromosomal imbalance levels to detect bladder cancer effectively.

**Methods:**

We enrolled 74 patients diagnosed with bladder cancer (BC), 51 healthy participants and 27 patients who were diagnosed with non-malignant urinary disease (UD). The Chromosomal Imbalance Analysis (CIA) was conducted in the tumours and urine of participants via the multiple annealing and looping-based amplification cycles-next-generation sequencing (MALBAC-NGS) strategy. The threshold of the CIA was determined with the receiver operating characteristic (ROC) curve. The comparison of the CIA with voided urine cytology was also performed in a subgroup of 55 BC patients. The consistency and discrepancy of the different assays were studied with the Kappa analysis and the McNemar test, respectively. The performance of the urinary CIA was also validated in an additional group of 120 BC patients, 15 UD and 45 healthy participants.

**Results:**

Good concordance (87.0%) in the assessments of patient tumour tissues and urine was observed. The urine-based evaluation also demonstrated a good performance (accuracy = 89.0%, sensitivity = 83.1%, specificity = 94.5%, NPV = 85.4% and PPV = 93.7%; AUC = 0.917, 95%CI =0.868–0.966, *P* < 0.001) in the training group, particularly in the patients with CIA-positive tumours (accuracy = 92.7%, sensitivity = 89.8%). The sensitivity and specificity in the validation group were 89.2 and 90.0%, respectively. Even in Ta/T1 and low-grade tumour patients, the sensitivity was 85–90%. The CIA also exhibited a significantly improved sensitivity compared to voided urine cytology.

**Conclusions:**

This is the first study employing the concept of whole genome imbalance combined with the MALBAC technique to detect bladder cancer in urine. MALBAC-CIA yielded significant diagnostic power, even in early-stage/low-grade tumour patients, and it may be used as a non-invasive approach for diagnosis and recurrence surveillance in bladder cancer prior to the use of cystoscopy, which would largely reduce the burden on patients.

**Electronic supplementary material:**

The online version of this article (10.1186/s12885-018-4571-7) contains supplementary material, which is available to authorized users.

## Background

Bladder cancer (BC) is one of the most common cancers in the world. Approximately 90% of patients with bladder cancer present with urothelial carcinoma, which has a high recurrence rate [1–3]. However, the diagnosis and the follow-up monitoring of BC has remained a challenge due to the lack of disease-specific symptoms [4]. Cystoscopy, although generally accepted as the gold standard for BC detection and surveillance, is an invasive procedure.

Voided urine cytology is a standard non-invasive approach adjunct to cystoscopy. The technique is highly specific (85–100%), but the sensitivity is tumour-grade dependent. Although good sensitivity was demonstrated for detecting high-grade urothelial cancer (80–90%, [1]), the technique is poor in terms of the detection of low-grade tumours, ranging from only 4–31% detection rates [4].

A profound number of new urinary biomarkers have been developed by laboratory and clinical investigations, many of which have also been approved by the FDA, such as NMP22, UroVysion® (fluorescence in situ hybridization, FISH) and BTAstat. Although many of these tests exhibit better sensitivity than urine cytology (up to 70–80%), they come with the price of lower specificity (median 70–85%) compared to cytology and therefore need to be further improved for wider application [4–7]. Therefore, a non-invasive, convenient and affordable urine-based test with high sensitivity and specificity is urgently in demand for BC diagnosis and monitoring.

Chromosomal instability is a common feature of tumour cells and has been reported to correlate with the development of bladder cancer [8]. Chromosomal instability might cause genomic abnormalities, such as alterations in chromosomal numbers and loss and/or gain of DNA in certain chromosomal segments [9, 10]. It has been recently reported that chromosome instability has the potential to function as a prognostic predictor in non-small-cell lung carcinoma [11]. In the present study, we developed a next-generation sequencing (NGS)-based evaluation approach, the chromosomal imbalance analysis (CIA), in combination with a previously reported whole genome amplification (WGA) technique of multiple annealing and looping-based amplification cycles (MALBAC) [12] to assess the chromosomal aberration level of cells in urine, and demonstrated the application of the MALBAC-CIA for BC detection.

## Methods

### Patient information

The study design is demonstrated in Additional file 1: Figure S1. In the training group, a total of 74 patients diagnosed with BC in Sun Yat-sen Memorial Hospital, Sun Yat-sen University were recruited from 2015 to 2016. We also enrolled 51 healthy participants and 23 patients who were diagnosed with non-malignant urinary disease (UD) as controls. For validation, 120 BC patients, 15 UD patients and 45 healthy participants from 2017 to 2018 were enrolled. Written informed consent was obtained from all participants, and the study was approved by the Medical Ethics Committee of Sun Yat-sen Memorial Hospital, Sun Yat-sen University. Tumour tissues were collected from 57 BC patients in the training group at the time of transurethral resection of bladder tumour (TURBT) or cystectomy and were stored at − 80 °C. The urine samples were collected from the second voided urine in the morning from all the participants, as shown in Additional file 1: Figure S1, and were stored at − 80 °C.

### Next-generation sequencing

DNA was extracted from the tumour tissues according to the manufacturer’s instructions (Qiagen, Germany). First, 50 ml of urine was centrifuged at 1600 g for 10 min to obtain cell pellets. The pellets were then washed and suspended in phosphate-buffered saline (PBS). Both the extracted DNA and the cell pellets were then subjected to NGS library preparation using the MALBAC-LIB kit (Yikon Genomics, China KT100800124) following the manufacturer’s instructions. The sequencing was performed on an Illumina HiSeq 2500 sequencer. Approximately 5 M sequencing reads were obtained from each sample.

### Chromosomal copy number variation (CNV) and CIA calculation

The adaptors and low-quality bases were removed from the raw data. High quality reads were mapped to the hg19 reference genome using BWA (version 0.7.12-r1039) with default parameters. Unique mapped reads were extracted from the alignment reads (.bam file). The whole reference genome was divided into non-overlapped observation windows (bins) with a size of 1000 Kb.

The relative copy number (xi) of each bin was calculated accordingly [12]. In brief, the read number and GC content were calculated in each bin. The bin read count was normalized based on the GC content and on a reference dataset to represent the relative copy number (xi). The R programming language was used to graph the xi of each bin to visualize copy number variations. Then, the Z value of each bin was calculated according to the formula:

$$ {\mathrm{Z}}_{\mathrm{i}}=\sqrt{\left| lo{g}_2\left(\frac{x_i}{2}\right)\right|} $$

The CIA score was calculated according to the formula:

$$ \mathrm{CIA}\ \mathrm{score}=\sum \limits_{\mathrm{i}={\mathrm{m}}_{\mathrm{b}}}^{{\mathrm{P}}_{\mathrm{b}}}\mid {\mathrm{Z}}_{\mathrm{i}}\mid $$

where mb and pb are the bins ranked m% and p%, respectively, according to the Z value (m = 95, *p* = 99).

### Voided urine cytology

Specimens from 57 of the 74 BC patients from the training group were prepared for urine cytology by the centrifuge and Cytospin methods. The slides were then stained and subjected to analysis by cytopathologists following the standard protocol. The evaluation of the test was recorded as negative, suspicious positive or positive.

### Statistical analysis

A receiver operating characteristic (ROC) curve was generated for the urine samples from BC patients and controls from the training group to determine the threshold of the CIA score and to evaluate its performance (Additional file 1: Figure S1). Fisher’s exact test was used to investigate the CIA score distribution in different T-stages and grades. The Kappa test was used to assess the consistency of CIA in the tissue and urine. The discrepancy from the various approaches was analysed by the McNemar test. All the analyses were performed with SPSS 22.0 (IBM, Chicago, IL, USA).

## Results

### The characteristics of the participants

A total of 74 patients diagnosed with BC were recruited in the training group, with a median age of 62. Of those, 57 patients provided tumour tissues, and all (74) provided a urine sample (Additional file 1: Figure S1). All 57 tissue and 71/74 urine samples generated qualified NGS data for the CIA analysis, among which 54 patients had tissue samples paired with urine samples. Fifty-one healthy participants and 23 UD patients provided urine samples for the subsequent CIA analysis as controls (Additional file 1: Figure S1). For the validation set, urine CIAs of an additional 120 BC patients, 15 UD patients and 45 healthy participants were calculated. The demographic and clinical information for all the participants, including the TNM stage and histologic grade, are summarized in Table 1, and the details are listed in Additional file 2: Tables S1-3.

**Table 1:** The demographic and clinical characteristics of the participants

	Training Group			Validation Group		
	BC	UD	NC	BC	UD	NC
Total No.	74	23	51	120	15	45
No. gender (%)						
Female	7 (9.5)	8 (34.8)	15 (29.4)	21(17.5)	5(33.3)	9 (20)
Male	67 (90.5)	15 (65.2)	36 (70.6)	99(82.5)	10(66.7)	36 (80)
Median age (range)	62 (28–90)	56 (18–72)	41 (23–69)	62(33–93)	54(22–73)	40(21–66)
Primary (%)	61 (82.4)	–	–	106(88.3)	–	–
Recurrent (%)	13 (17.6)	–	–	14(11.7)	–	–
No. tumour stage (%)						
Tis	1 (1.4)	–	–	2(1.7)	–	–
Ta	23 (31.1)			34(28.3)		
T1	24 (32.4)			61(50.8)		
T2	16 (21.6)			13(10.8)		
T3	5 (6.8)			10(8.3)		
NA	5 (6.8)			0(0.0)		
No. tumour grade (%)						
PUNLMP	5(6.8)	–	–	0(0.0)	–	–
LG	9(12.2)			33(27.5)		
HG	59 (79.7)			83(69.2)		
NA	1 (1.4)			4(3.3)		

*BC* bladder cancer patients, *UD* non-malignant urinary disease patients, *NC* healthy participants

### The cut-off determination and distribution of CIA

**Fig. 1:**
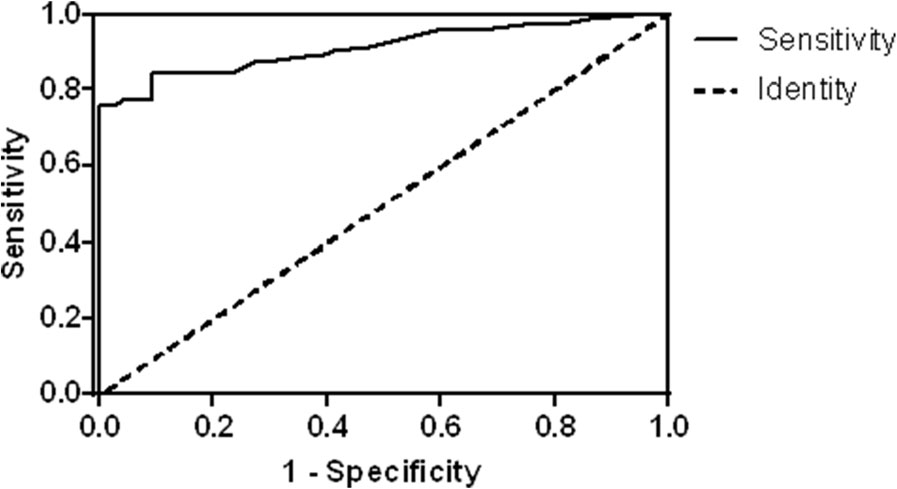
ROC curve analysis for urine CIA scores. To determine the best cut-off value that discriminated between malignant BC patients and control groups, urine CIA scores from 71 BC, 23 UD and 51 healthy participants were included. The cut-off was defined as 24 [Accuracy = 89.0%, sensitivity = 83.1%, specificity = 94.5%, NPV = 85.4% and PPV = 93.7%]. Area under the curve (AUC) =0.917, 95%CI =0.868–0.966, *P* < 0.001

The cut-off of CIA was determined by performing the ROC curve on all 145 urine samples from the training group (71 BC, 23 UD and 51 healthy participants, Additional file 1: Figure S1). A cut-off of 24 was ultimately determined (Fig. 1), which yielded an accuracy of 89.0% and an area under the curve (AUC) of 0.917 (95%CI =0.868–0.966, *P* < 0.001). The sensitivity and the specificity were 83.1 and 94.5%, respectively. The negative predictive value (NPV) and the positive predictive value (PPV) were 85.4 and 93.7%, respectively.

**Fig. 2:**
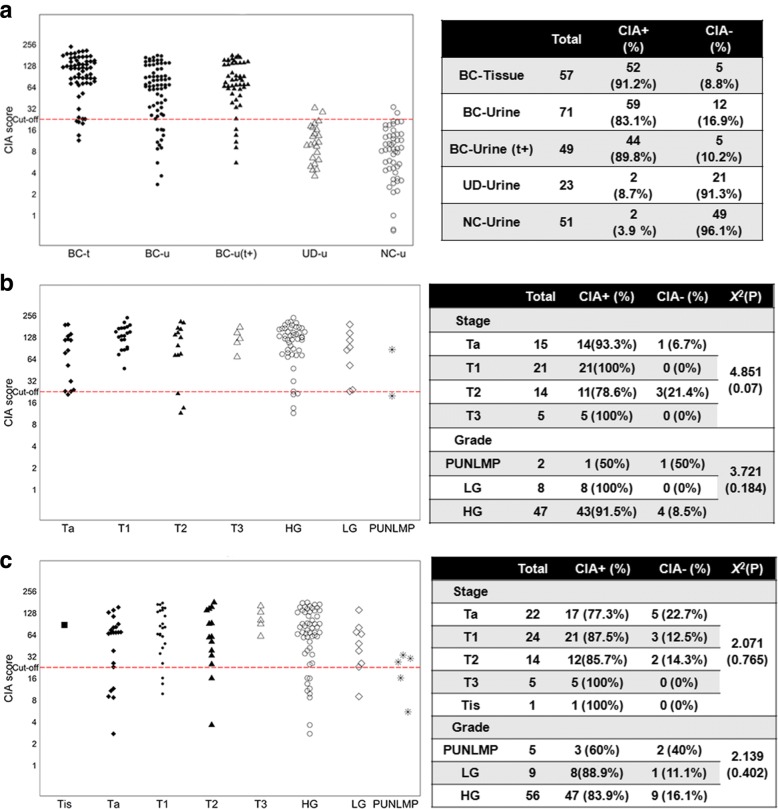
The distribution of CIA scores. **a** CIA scores in tumour tissues from bladder cancer patients (BC-t), urine of BC patients (BC-u), urine of BC patients with paired CIA positive tumour tissues (BC-u (t+)), urine of non-malignant urinary disease patients (UD-u) and healthy controls (NC-u); **b** Tumour tissue CIA scores in different stages and grades; **c** Urine CIA scores in different stages and grades. The cut-off for positive CIA definition was set to 24. The *P* value was calculated from Fisher’s Exact Test

As illustrated in Fig. 2a, the BC patients were distinguished from the healthy participants and the UD patients by the urine CIA score. In particular, 83.1% (59/71) of the BC patients had CIA scores above the cut-off of 24. On the other hand, the scores of the healthy control and UD patients were mainly clustered under 24, with only 3.9% (2/51) of the former group and 8.7% (2/23) of the latter group being greater than the cut-off. In the 57 tumour tissues, 52 (91.2%) had CIA scores above the cut-off of 24 (Fig. 2a), and 3 of them failed to generate paired urine outcomes (Additional file 2: Table S1). In the remaining 49 tumour CIA + patients, concordant positive urine CIA scores were observed in 44 (89.8%).

The distribution of tissue and urine CIA scores in different TNM stages and grades are displayed in Fig. 2b, c. No significant differences were observed for CIA-positive and negative proportions among the various stages or grades (*P* > 0.05).

### The concordance of assessment between urine and tumour tissues via CIA

**Fig. 3:**
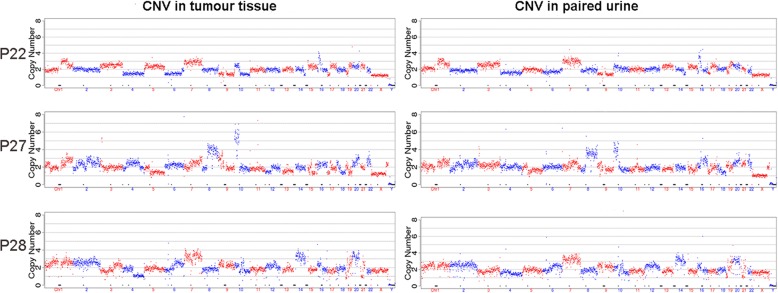
The demonstration of chromosomal CNV patterns in BC patients. The CNV profiles in tumour tissue and paired urine samples for patients No. 22, No. 27 and No. 28 (See Additional file 2: Table S1) are shown

The chromosomal CNV profiles were investigated in the urine and paired tumour tissues of BC patients. The results showed similar CNV patterns in the urine and tissue of the same patient but diverse profiles among different individuals (Fig. 3). Then, the CIA scores were compared between the two types of samples. In 54 patients whose tumour tissues and paired urine were provided, 3 (5.6%) were CIA-negative and 44 (81.5%) were identified as CIA-positive in both urine and tissue samples (Table 2). Seven patients (13.0%) had inconsistent outcomes between urine and tissue samples. The concordance rate was calculated as 87.0% (Kappa = 0.392, *P* = 0.003), and no significant discrepancy in the evaluation was detected between the two types of samples, according to the McNemar Test (*P* = 0.453).

**Table 2:** Concordance of CIA evaluation between tumour tissues and urine samples. (*N* = 54)

CIA in urine	CIA in tissue		Total (%)
	Negative (%)	Positive (%)	
Negative (%)	3 (5.6%)	5 (9.3%)	8 (14.8%)
Positive (%)	2 (3.7%)	44 (81.5%)	46 (85.2%)
Total (%)	5 (9.3%)	49 (90.7%)	54 (100%)
Concordance (%)	87.0%		
Kappa	0.392, *P* = 0.003		
McNemar Test	*P* = 0.453		

### Screening of BC patients by urine CIA score

The performance of the CIA scores as a marker to distinguish BC patients from healthy participants and UD patients was assessed and is shown in Table 3. The overall sensitivity and accuracy were 83.1 and 89.0%, respectively. Interestingly, when we limited the BC patients into the subgroup with CIA-positive tissues, the sensitivity was improved to 89.8%. The accuracy also increased to 92.7%.

**Table 3:** The performance of the urine CIA score in training group to distinguish BC patients in different TNM stages and histology grades

BC patients	No. of (Case/Control)	Sensitivity	Specificity	Accuracy
All patients	71/74	83.1%	94.5%	89.0%
Tis + Ta + T1	47/74	83.0%	94.5%	90.1%
T2 + T3	19/74	94.7%	94.5%	94.6%
PUNLMP	5/74	60%	94.5%	92.4%
LG	9/74	88.9%	94.5%	94.0%
HG	55/74	83.9%	94.5%	90.0%
With CIA+ Tissues	49/74	89.8%	94.5%	92.7%
Ta + T1	34/74	88.2%	94.5%	92.6%
T2 + T3	14/74	100%	94.5%	95.5%
PUNLMP	1/74	0%	94.5%	93.3%
LG	8/74	87.5%	94.5%	93.9%
HG	40/74	92.5%	94.5%	93.9%

Furthermore, we investigated the performance of CIA in various subgroups of BC patients with different TNM stages and histology grades (Table 3). The performance in muscle-invasive BC (T2 and T3) showed higher sensitivity than in non-muscle-invasive BC (Ta and T1). Notably, in the subset of patients with CIA-positive tissues, the sensitivity approached 90% for Ta + T1 and was 100% for T2 + T3 in our cohort. Regarding the histological grade, the CIA resulted in higher sensitivity in histologically low-grade (LG) than histologically high-grade (HG) patients (88.9% vs. 83.9%). However, the trend was the opposite in the subgroup of tissue CIA + patients (HG vs. LG: 92.5% vs. 85.7%). This discrepancy might be attributed to the limited number of LG patients enrolled in the study. In the 5 papillary urothelial neoplasms of low malignant potential (PUNLMP) patients, the sensitivity of CIA was not satisfying (60%).

### The comparison of the CIA with voided urine cytology

Voided urine cytology was also conducted in 57 BC patients from the training group. Among these patients, the urine CIA analysis was successfully achieved in 55 patients, and the results were compared (Table 4). Only 29 (52.7%) of the BC patients were identified as positive by cytology, whereas 44 (80%) urine samples were detectable by CIA. This improvement was significant (*p* = 0.004).

**Table 4:** The comparison of the CIA results with voided urine cytology in BC patients

Urine CIA	Cytology		Total (%)
	Negative (%)	Positive (%)	
Negative (%)	6 (10.9%)	5 (9.1%)	11 (20.0%)
Positive (%)	20 (36.4%)	24 (43.6%)	44 (80.0%)
Total (%)	26 (47.3%)	29 (52.7%)	55 (100%)
McNemar Test	*P* = 0.004		

The original cytology results included negative, suspicious positive and positive. The results of suspicious positive and positive were regarded as positive in this comparison

### The validation of CIA score in detecting BC patients

To validate the established CIA score in discriminating BC patients, a validation cohort of 120 BC patients and 60 control participants was recruited. The CIA resulted in a sensitivity of 89.2% and a specificity of 90.0% in the validation set (Table 5). Particularly, the sensitivity was 83.3% in Tis/Ta patients, and it increased to 88.5 and 100% in T1 and T2/T3 patients, respectively. In terms of tumour grade, HG patients showed a higher sensitivity compared to LG patients (90.4% vs. 84.8%).

**Table 5:** The validation of the urine CIA score to distinguish BC patients

BC patients	No. of (Case/Control)	Sensitivity	Specificity	Accuracy
All patients	120/60	89.2%	90.0%	89.4%
Tis + Ta	36/60	83.3%	90.0%	87.5%
T1	61/60	88.5%	90.0%	89.3%
T2 + T3	23/60	100.0%	90.0%	92.8%
LG	33/60	84.8%	90.0%	88.2%
HG	83/60	90.4%	90.0%	90.2%

## Discussion

Chromosomal aberration is a common occurrence in tumours. In addition to aneuploidy, alterations in chromosomal architecture, focal amplifications and deletions are observed in cancer genomes. As a driving factor, the chromosomal abnormality manifests at the earliest stages of tumourigenesis and accumulates throughout subsequent tumour development [9, 13–15]. The urinary FISH test (UroVysion®), which probes alterations in chromosomes 3, 7, 17 and 9p21, is one of the commercially available urinary biomarkers used to detect BC. The sensitivity and specificity have been reported in systematic reviews and meta-analyses to exceed 70% and ~ 80%, respectively, but with a broad range among different studies [16–18]. The methylation patterns of a number of candidate genes have also been explored as potential biomarkers [16, 19]. More recently, a combination of methylation status of TWIST, ONECUT2, and OTX1 with mutational analyses of FGFR3, TERT, and HRAS has been reported to detect bladder cancer with a sensitivity of 97% and a specificity of 83% [20, 21]. However, due to the diversity of the tumourigenesis driver mutations and the randomness of the somatic passenger mutations [22], tremendous genetic heterogeneity is spatially and temporally observed in tumour cells and is expected in different individuals, as is the case in bladder cancer [23] (Fig. 3). Conceivably, using the manifestation of genomic abnormality/imbalance that comprehensively assesses the variation across the whole genome as an indicator of bladder cancer detection might show superior sensitivity. Previously, different evaluation scores based on whole-genome sequencing were reported in prostate, colorectal and breast cancers for diagnosis and prognosis among limited numbers of patients/controls [9, 24–26]. It is known that the number of exfoliated tumour cells varies in BC patients. This amount may be associated with the size and grade of the tumour. Therefore, an approach capable of detecting a small number of exfoliated tumour cells is in demand for urine-based diagnosis. As a commonly used WGA technique for single cell genome studies, MALBAC facilitates the analysis of trace amounts of starting materials and does not require additional DNA extraction [12, 27]. MALBAC possesses the advantages of convenience and rapidness compared to a routine library construction process for NGS.

In the present study, we developed a novel strategy based on NGS that incorporates MALBAC and a new chromosomal imbalance evaluation approach, CIA, to assess the aberrant level of the chromosomal genome, and we demonstrated its application in detecting BC for the first time. Approximately 92% of the BC patients were identified as positive in tumour tissues according to the CIA, regardless of the TNM stage and histological grade, indicating its potentially wider utility for diagnosis. Moreover, as demonstrated in Fig. 3, the CNV profile of urine shows characteristics similar to tissues derived from the same patient, suggesting that urine cell pellets are representative of tumours for CNV assessment. The urine CIA also exhibits concordance with tissue CIA, indicating its potential as a non-invasive diagnostic strategy (Table 2). Notably, the performance of urine CIA was superior in the subgroup of patients carrying CIA-positive tumours than in all the patients. Tissue CIA might serve as a prior test to select patients possessing positive CIA scores in primary tumours for the subsequent recurrence surveillance by non-invasive urine CIA. Nonetheless, the heterogeneity in primary and recurrent tumours should also be taken into account, and the feasibility of this method needs to be investigated and validated by prospective studies.

The performance of this technique was also compared with other non-invasive methods. Voided urine cytology is routinely used in the clinic with good specificity (> 90%); however, the sensitivity has been reported to be 30–50% [4]. CIA showed significantly improved sensitivity in detecting BC patients in this study (80–90% vs. 52.7%), which is also superior to the reported cytology sensitivity in the literature. The FISH (UroVysion®) probes alterations in chromosomes 3, 7, 17 and 9p21 and the sensitivity and specificity have been reported to be ~ 50–80% and 70–85%, respectively [4, 17, 18]. CIA displayed a superior performance, with both the sensitivity and specificity being ~ 90% in the training and validation groups. Other commercially available markers (such as NMP22, ImmunoCyt and BTA stat) also show unsatisfactory performance [4, 16].

One major limitation of the currently available urinary biomarkers is the poor sensitivity for early-stage and lower-grade tumours [1, 4, 18]. However, 30–80% of patients diagnosed with a low-grade Ta/T1 primary tumour undergo recurrence within 5 years [1–3]. In addition, tumours generally are larger or in a more advanced stages at diagnosis than during surveillance. A non-invasive test with high sensitivity, particularly for early-stage and low-grade tumours, is important for the surveillance of patients by reducing the use of invasive tests such as cystoscopy and thereby improving the patient quality of life [1]. However, it has been reported that cells from men with low-grade BC accumulated fewer CNVs than those from cases with high-grade cancer [28]. Hurst et al. [29] also discovered that the more genomically unstable subtype of Ta bladder cancer was distinguished by loss of chromosome 9q, and the other subtype contained no or few copy-number alterations. This outcome might explain the observation that the CIA showed a slightly lower sensitivity in early-stage and low-grade tumours (83–85%) than that of more advanced stage and high-grade tumours (90–100%). However, the observed sensitivity is better than the performance of other commercial markers in the same stage/grade, which have been reported to be less than 80% in most cases [1, 4, 18]. The sensitivity of CIA was observed to be only 60% in the 5 PUNLMP patients, which might be explained by its low level of malignancy. However, a limited number of patients were included in the present study; therefore, a conclusive statement cannot be made without further validation in a larger set of patients.

The cost of this new technique is estimated to be $200–300 per patient. Although this is relatively costly compared to the methylation and mutation combination assay reported recently ($23) [21], with the rapid reductions in NGS cost, the CIA assay is expected to become cost effective in the near future. Due to the utility of MALBAC technology, the CIA assay does not require a large amount of DNA, which makes it a more suitable technique for urine-based testing. Compared to the DNA methylation assay, the minimum DNA input of which is approximately 50 ng (the amount present in 8000 cells), the MALBAC assay is applicable to a single cell equivalent amount of DNA [12, 27].

It has been reported that genetic mutations in certain genes, such as FGFR3, RB1, HRAS, TP53, TSC1, TERT and others, occur in urinary bladder tumours [30]. A proportion of urothelial tumours also harbour mutations that are potentially therapeutic targets, including the FGFR3, TSC1 and PIK3CA mutations [31–34]. It is quite possible that a combination of the reported CIA and other mutations might improve the detection rate of bladder cancers and might be informative for therapy selection.

## Conclusion

Overall, we developed a new strategy based on the chromosomal imbalance/aberration level and demonstrated its application in BC detection for the first time. Good concordance (87.0%) in the assessments obtained from patient tumours and urine was observed. The urine-based evaluation also demonstrated a good performance (accuracy = 89.0%, sensitivity = 83.1%, specificity = 94.5%, NPV = 85.4% and PPV = 93.7%; AUC = 0.917, 95%CI =0.868–0.966, *P* < 0.001) in the training group, particularly in patients with CIA-positive tumours (accuracy = 92.7%, sensitivity = 89.8%). The performance was also validated in an additional group, with a sensitivity and specificity of ~ 90%. It is conceivable that the present approach might have the potential to be a non-invasive test for BC diagnosis and to be subsequent surveillance prior to cystoscopy use. We envision that prospective cohort studies, with larger samples incorporating both BC patients and a certain percentage of patients with related symptoms and/or signs, will be designed to further validate the feasibility of monitoring bladder cancer patients.

## Additional files

Additional file 1: **Figure S1.** The illustration of the study design. The square (□) represents bladder cancer patients. The circle (○) and triangle (**▲**) represent the healthy participants and patients diagnosed with non-malignant urinary diseases, respectively. The number refers to the number of participants (TIF 1991 kb).

Additional file 2: The demographic and clinical characteristics of training group. **Table S1.** The demographic and clinical characteristics of bladder cancer (BC) patients of training group. The originally reported cytology classification included negative (−), suspicious (+−) and positive (+). **Table S2.** The demographic and clinical characteristics of non-malignant urinary disease (UD) patients of training group. **Table S3.** The demographic characteristics of healthy controls of training group (XLSX 27 kb).

## Abbreviations

CIA: Chromosomal Imbalance Analysis
BC: bladder cancer
NGS: next-generation sequencing
WGA: whole genome amplification
MALBAC: looping-based amplification cycle
UD: non-malignant urinary disease
CNV: Chromosomal copy number variation
ROC: receiver operating characteristic
TURBT: transurethral resection of bladder tumour
PBS: phosphate buffered saline
AUC: area under the curve
NPV: negative predictive value
PPV: positive predictive value
LG: low-grade
HG: high-grade
PUNLMP: papillary urothelial neoplasms of low malignant potential
FISH: fluorescence in situ hybridization
