# Supplementary figures and images for: MALBAC-based chromosomal imbalance analysis: a novel technique enabling effective non-invasive diagnosis and monitoring of bladder cancer
Source: BMC Cancer. 2018 Jun 15;18:659. doi: 10.1186/s12885-018-4571-7 (PMC6003132; doi:10.1186/s12885-018-4571-7)

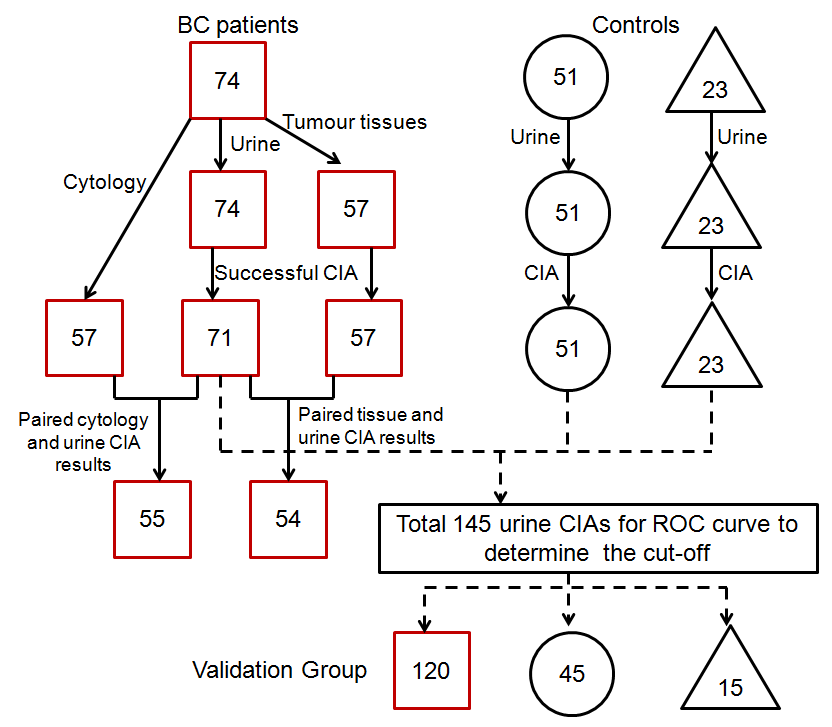

Supplement: Supplementary file 1 — Figure S1. The illustration of the study design. The square (□) represents bladder cancer patients. The circle (○) and triangle (▲) represent the healthy participants and patients diagnosed with non-malignant urinary diseases, respectively. The number refers to the number of participants (TIF 1991 kb). [file 12885_2018_4571_MOESM1_ESM.tif]
